# A Membrane‐Permeable and Immobilized Metal Affinity Chromatography (IMAC) Enrichable Cross‐Linking Reagent to Advance In Vivo Cross‐Linking Mass Spectrometry

**DOI:** 10.1002/anie.202113937

**Published:** 2022-01-27

**Authors:** Pin‐Lian Jiang, Cong Wang, Anne Diehl, Rosa Viner, Chris Etienne, Premchendar Nandhikonda, Leigh Foster, Ryan D. Bomgarden, Fan Liu

**Affiliations:** ^1^ Department of Structural Biology Leibniz-Forschungsinstitut für Molekulare Pharmakologie im Forschungsverbund Berlin e.V. (FMP) Campus Berlin-Buch Robert-Roessle-Str. 10 13125 Berlin Germany; ^2^ Thermo Fisher Scientific 355 River Oaks Pkwy San Jose CA 95134 USA; ^3^ Thermo Fisher Scientific 3747N Meridian Rd Rockford IL 61101 USA; ^4^ PCharité—Universitätsmedizin Berlin Charitépl. 1 10117 Berlin Germany

**Keywords:** Cross-linking, Mass spectrometry, Protein-protein interactions, Protein structures, Proteomics

## Abstract

Cross‐linking mass spectrometry (XL‐MS) is an attractive method for the proteome‐wide characterization of protein structures and interactions. Currently, the depth of in vivo XL‐MS studies is lagging behind the established applications to cell lysates, because cross‐linking reagents that can penetrate intact cells and strategies to enrich cross‐linked peptides lack efficiency. To tackle these limitations, we have developed a phosphonate‐containing cross‐linker, tBu‐PhoX, that efficiently permeates various biological membranes and can be robustly enriched using routine immobilized metal ion affinity chromatography. We have established a tBu‐PhoX‐based in vivo XL‐MS approach that enables cross‐links in intact human cells to be identified in high numbers with substantially reduced analysis time. Collectively, the developed cross‐linker and XL‐MS approach pave the way for the comprehensive XL‐MS characterization of living systems.

## Graphical abstract

A phosphonate‐containing cross‐linker, tBu‐PhoX, has been developed that efficiently permeates various biological membranes and can be robustly enriched using routine immobilized metal ion affinity chromatography (IMAC). The optimized tBu‐PhoX‐based in vivo cross‐linking mass spectrometry (XL‐MS) system enables the time‐efficient and detailed characterization of protein interaction landscapes in intact human cells.

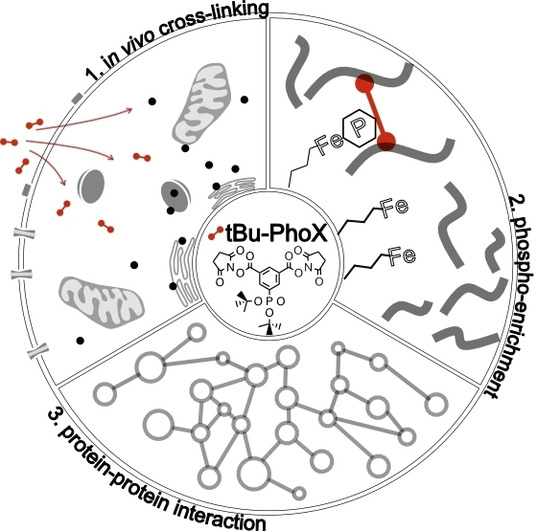

The cellular proteome is organized through extensive networks of noncovalent interactions—ranging from stably bound macromolecular assemblies to transiently formed complexes that associate and dissociate rapidly during cellular signalling.^[1]^ Characterizing these protein–protein interactions (PPIs) is crucial to understand the regulatory mechanisms of the cell. A powerful approach to systematically study cellular PPIs is cross‐linking mass spectrometry (XL‐MS). In XL‐MS, native protein contacts are covalently captured using a cross‐linker, a small organic molecule composed of a spacer arm and two functional groups that are reactive toward specific amino acid side chains. After proteolytic digestion of the cross‐linked sample, cross‐links between amino acids can be localized by MS‐based peptide sequencing. Since cross‐linkers have a defined maximum length, detected cross‐links reveal maximum distances of amino acids within or between proteins. This information provides insights into protein conformations, structures, and interaction networks.

Although initially limited to purified protein assemblies, XL‐MS can now be applied to complex biological systems. This was made possible through the development of advanced cross‐link search engines,^[2]^ sample preparation strategies,^[3]^ and cross‐linker designs.^[4]^ In particular, several proteome‐wide XL‐MS studies in lysed and intact cells have shown that cross‐link identification can be improved by enabling cross‐linkers to be enriched, for example, by adding biotin or azide/alkyne tags to allow affinity‐ or click‐chemistry‐based enrichment of cross‐linker‐modified peptides from the large excess of linear peptides in the digestion mixture.^[5]^ Recently, a phosphonic acid based cross‐linker, PhoX, was introduced as a highly efficient and specific alternative to the existing biotin‐ or azide/alkyne‐tagged reagents.^[6]^ PhoX enables cross‐link enrichment by immobilized metal ion affinity chromatography (IMAC), a fast and extremely robust enrichment strategy well established in various areas of protein mass spectrometry. However, although PhoX has proven useful for cross‐link identification from cell lysates, it cannot permeate the cell membrane and is, therefore, unsuitable for in vivo XL‐MS.

Here, we set out to develop a phosphonic acid based cross‐linker that is compatible with in vivo XL‐MS. We hypothesized that the membrane permeability of PhoX is impaired by the negative charges of its phosphonate moieties. Therefore, we developed tBu‐PhoX, a cross‐linker in which the phosphonic acid hydroxy groups are protected by *t*‐butyl groups to mask the negative charges (Scheme 1). To examine the membrane permeability of tBu‐PhoX, we cross‐linked various membrane‐enclosed biological systems, including human HEK293T cells, mitochondria isolated from mouse hearts, and gram‐positive *B. subtilis*, and monitored the shift of protein bands on SDS‐PAGE (Figure 1, Figure S1). In all these systems, we observed concentration‐dependent migration of proteins to higher molecular weights for cross‐linker concentrations of 0.5 and 1.0 mM, indicating efficient membrane permeation and cross‐linking. At higher cross‐linker concentrations, we still observed high molecular weight species, but the protein bands became fainter, suggesting an increased formation of covalently linked complexes that are too large to enter the gel. By contrast, applying PhoX to intact HEK293T cells resulted in a band pattern identical to the non‐cross‐linked control. Furthermore, we found that the membrane permeability of tBu‐PhoX was reduced for gram‐negative *E. coli* bacteria, likely due to the characteristics of the gram‐negative cell wall (Figure S1C).

**Scheme 1:**
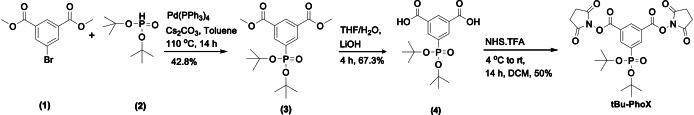
Synthesis of tBu‐PhoX (*tert*‐butyl disuccinimidyl phenyl phosphonate).

**Figure 1:**
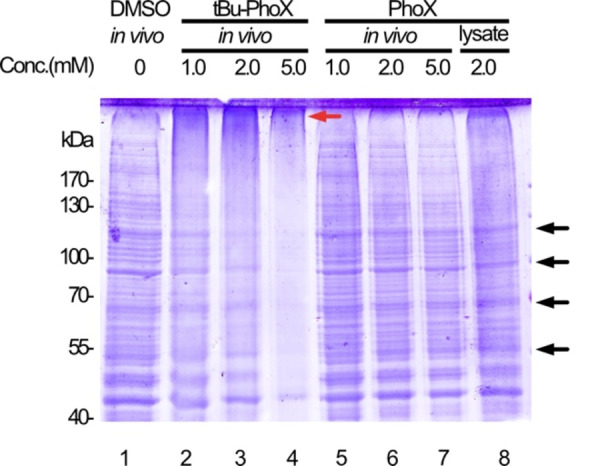
SDS‐PAGE of HEK293T cells in vivo cross‐linked with either PhoX or tBu‐PhoX at different cross‐linker concentrations. Black arrows indicate gel bands that disappear after efficient protein cross‐linking. The red arrow indicates the accumulation of proteins with higher molecular weights.

Having shown that tBu‐PhoX permeates various biological membrane systems, we next developed a tBu‐PhoX‐based in vivo XL‐MS workflow that increases the speed and efficiency of sample processing and cross‐link enrichment compared to previous proteome‐wide XL‐MS strategies (Figure 2). First, cross‐linked proteins are digested into peptides following a standard protein digestion procedure (see Experimental Section for details). Second, the digestion mixture is subjected to a pre‐clearance step using IMAC beads to remove the endogenous modified (especially phosphorylated) peptides that would interfere with crosslink enrichment. Third, the flow‐through of the pre‐clearance step is incubated in dilute trifluoroacetic acid (TFA) solution to remove the *t*‐butyl groups and expose the phosphonic acid group for a secondary IMAC enrichment. Fourth, cross‐links are enriched using a standard IMAC procedure and finally analyzed by LC‐MS for cross‐link identification (see Experimental Section for details). Importantly, this two‐stage IMAC procedure, made possible by initially protecting the phosphonate groups in tBu‐PhoX, obviates the hours‐long phosphatase treatment required in the original PhoX workflow to remove contaminating phosphorylated peptides prior to cross‐link enrichment.^[6]^ Replacing phosphatase pre‐treatment with IMAC pre‐clearance shortens the sample preparation time to 30 minutes. Moreover, it may allow removal of unwanted species that can be captured by IMAC in addition to phosphorylated peptides (e.g. oligonucleotides or N‐glycosylated peptides).^[7]^

**Figure 2:**
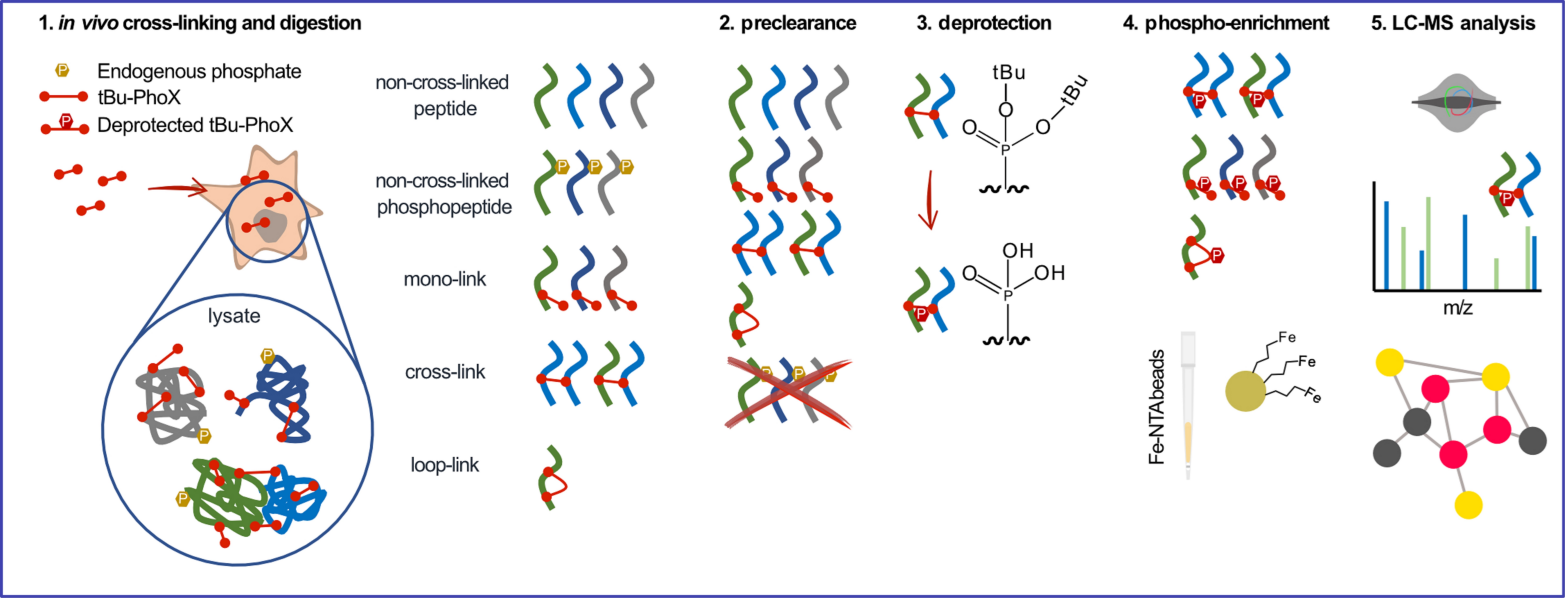
The conceptual workflow of in vivo cross‐linking with tBu‐PhoX and subsequent sample processing.

Next, we optimized several analytical parameters of our in vivo XL‐MS workflow to maximize cross‐link detection. First, we assessed the efficiency of phosphopeptide removal by pre‐clearance using IMAC beads. In this experiment, we cross‐linked intact HEK293T cells using tBu‐PhoX, digested the cross‐linked proteins, and applied a pre‐clearance IMAC step to remove endogenous phosphopeptides. After deprotection, cross‐links were enriched by a second IMAC step. We measured the number of phosphopeptides and cross‐links in the IMAC eluates by a single‐shot 120 min LC‐MS run and found only hundreds of phosphopeptides from the second IMAC but 4128 phosphopeptides from pre‐clearance IMAC (Figure S2A). This highlights the efficiency of removing phosphopeptides by the pre‐clearance IMAC step. Moreover, we identified 22 % more cross‐links (1165 versus 952 cross‐links) by using the workflow with pre‐clearance IMAC compared to a single‐stage IMAC protocol, demonstrating the benefits of removing interfering modified peptides when using IMAC‐enrichable cross‐linkers (Figure 3A). Importantly, we only identified 28 cross‐links from the eluate of the pre‐clearance IMAC, indicating that the loss at this stage is negligible (ca. 2.4 %; Figure 3A).

**Figure 3:**
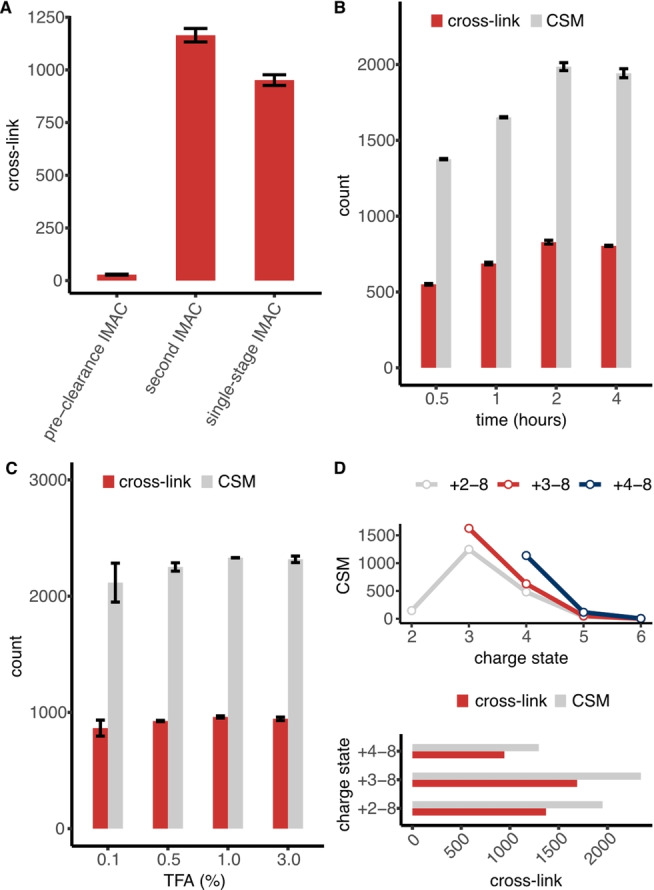
Optimization of sample processing and LC‐MS parameters. A) Number of cross‐links identified from different IMAC elution steps. B, C) Number of cross‐links and CSMs identified using different: B) incubation times (keeping TFA fixed at 0.5 %) and C) TFA concentrations (keeping the incubation time fixed at 2 h) for tBu‐PhoX deprotection. D) Number of cross‐links and CSMs identified when different charge states were included in the corresponding LC‐MS measurements.

We then investigated the efficacy of phosphonate deprotection at the peptide level. We prepared in vivo cross‐linked HEK293T samples using tBu‐PhoX and analyzed how the number of cross‐links after deprotection changes on varying the acidity (TFA concentration) and incubation time. The number of cross‐links was similar at different concentrations of TFA and reached a maximum after two hours of incubation time. For simplicity of handling (i.e. keeping a relatively low sample volume for the following IMAC enrichment step), we opted for a two‐hour deprotection step in 0.5 % TFA (Figure 3B, C).

Then we tested how cross‐link identifications are influenced by different acquisition parameters of our Orbitrap Tribrid mass spectrometer, namely charge state selection and the compensation voltages (CVs) applied in high‐field asymmetric‐waveform ion‐mobility spectrometry (FAIMS). We identified the highest number of tBu‐PhoX cross‐linked peptides when considering charge state +3 and higher (Figure 3D, S2B). All tested FAIMS CV settings provided a similar number of cross‐links (Figure S3A–C). Therefore, we selected the 3‐CV combination (−50/−60/−70 V), which yielded the most cross‐link spectrum matches (CSMs) for subsequent LC‐MS measurements (Figure S3D).

We applied our in vivo XL‐MS workflow with optimized parameters to intact HEK293T cells. To ensure data quality, we performed a cell viability assay before and after cross‐linking and observed only a slight decrease in the cell viability after cross‐linking (89 % before and 80–83 % after; Figure S4). We identified 3103 cross‐links (6845 CSMs) in a single‐shot 180 min LC‐MS measurement (Figure S5). Remarkably, 99.8 % of the identifications were phospho‐attached (i.e. mono‐links, loop‐links, and cross‐links), underscoring the highly efficient IMAC enrichment and removal of non‐cross‐linked peptides. However, it is well‐established that enrichable cross‐linkers are unable to separate interpeptide cross‐links from cross‐linker‐modified linear peptides (mono‐links and loop‐links). This is confirmed by our data, in which cross‐links make up only one sixth of the total identifications, with mono‐links and loop‐links being dominant (Figure S5). These unwanted species can be efficiently removed by size‐exclusion chromatography (SEC) to further improve cross‐link identification.^[5a]^ Therefore, we subjected the IMAC‐enriched sample to SEC fractionation and collected five early fractions with a high relative abundance of cross‐linked peptides for LC‐MS analysis (Figure S6). Using a 180 min LC gradient and the optimized analytical parameters, we obtained a total number of 9547 cross‐links from in vivo tBu‐PhoX cross‐linked HEK293T cells (Figure 4A). SEC fractionation increased the number of cross‐links almost threefold and covers 96 % of the cross‐links that were identified in the single‐shot measurement (Figure S7A). Gene ontology analysis shows that the cross‐linked proteins are involved in a wide range of molecular functions, biological processes, and cellular components, thus indicating that tBu‐PhoX can reveal PPIs in all cellular regions (Figure S7B).

**Figure 4:**
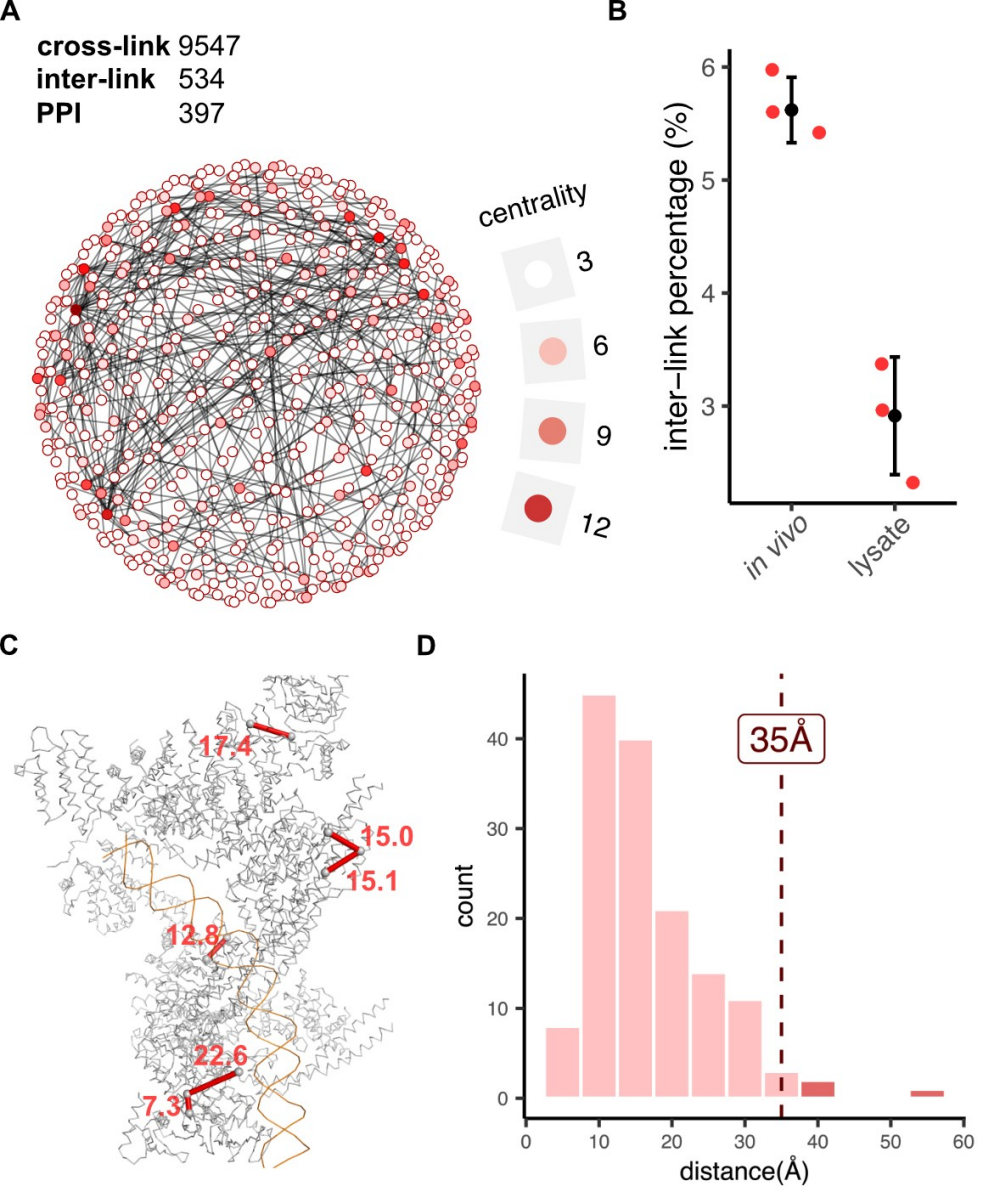
In vivo cross‐linking of intact HEK293T cells using tBu‐PhoX. A) Network plot of all detected PPIs. Proteins are depicted as dots colored according to their centrality, which is a measure of the influence of an individual protein in a network. B) Number of interprotein cross‐links identified by in vivo and cell lysate cross‐linking. Error bars represent the standard deviation of three biological replicates. C) Mapping cross‐links onto the high‐resolution structure of a cohesin complex (PDB awg3). D) Histogram of C_α_‐C_α_ distances of cross‐linked residues in the protein complexes shown in Figures 4C and S8.

Furthermore, we queried whether in vivo XL‐MS of intact cells captures a different PPI landscape than XL‐MS of cell lysates. To test this, we prepared tBu‐PhoX cross‐linked lysates from HEK293T cells and processed the sample using the same workflow as the in vivo XL‐MS experiment. We identified 9393 cross‐links from five SEC fractions. This suggests that tBu‐PhoX allows lysate and in vivo XL‐MS to be performed with comparable efficiency, whereas several previous XL‐MS studies reported a substantial reduction of cross‐link coverage in vivo.[^5d^, ^5e^, ^8^] Comparing our in vivo and lysate data shows that the number of interprotein cross‐links is higher in the in vivo XL‐MS experiment, which results in a more interconnected PPI network (Figure 4B). This effect may be explained by the crowded nature of the cellular environment, in which proteins are tightly packed and engage in a multitude of interactions that are partially disrupted by cell lysis and dilution. We visualized 145 in vivo detected cross‐links on known 3D structures of 8 selected protein complexes (Figure 4C, Figure S8). We observed that 96.6 % of the cross‐links are within the maximum distance constraint of 35 Å (20 Å from the C_α_‐C_α_ distance based on the rigid structure of PhoX cross‐linker^[6]^ plus 15 Å of protein flexibility, Figure 4D), supporting the applicability of our XL‐MS workflow for the in vivo structural analysis of endogenous protein complexes.

Lastly, we compared the performance of the tBu‐PhoX with PhoX in the characterization of PPI networks of cell lysates. We prepared PhoX cross‐linked lysates from HEK293T cells using the same cross‐linking condition as in the aforementioned lysate cross‐linking experiment with tBu‐PhoX. To remove endogenous phosphopeptides, digested peptides were treated with alkaline phosphatase for two hours before a single‐stage IMAC enrichment. LC‐MS analyses were performed using the same LC‐MS method as for tBu‐PhoX. This experiment yielded 2117 cross‐links, which is slightly higher than the number of cross‐links identified using tBu‐PhoX (1942 cross‐links, Figure S9). However, the PhoX‐based XL‐MS pipeline requires a longer sample preparation time because of the alkaline phosphatase retreatment and an extra desalting step afterwards.

Taken together, we have developed and applied the new enrichable and membrane permeable cross‐linker tBu‐PhoX for in vivo XL‐MS. tBu‐PhoX efficiently penetrates various biological membranes under widely used cross‐linking conditions (cross‐linker concentration of 1–5 mM), thus allowing protein cross‐linking in intact organelles and living cells. The *t*‐butyl groups on tBu‐PhoX enable a highly efficient two‐stage IMAC sample preparation procedure; first, making the cross‐linker inert to IMAC to facilitate fast and thorough IMAC‐based extraction of unwanted phosphorylated peptides, then—through removal of the *t‐*butyl groups—exposing a phosphonic acid group for efficient secondary IMAC enrichment of cross‐linker‐modified peptide species. By subsequent SEC fractionation, cross‐links can be further enriched for LC‐MS analysis.

XL‐MS plays an increasingly important role in characterizing protein structures and interactions in living systems.^[9]^ To foster this development, efficient in vivo XL‐MS approaches are urgently required. The in vivo XL‐MS workflow reported here addresses this need, providing a similar cross‐link identification capacity as published lysate‐based XL‐MS studies but requiring less than one tenth of the previously reported measurement times (15 h in our study compared to an average of 240 h in other proteome‐wide XL‐MS studies using non‐enrichable cross‐linkers, Table S1). This result highlights that tBu‐PhoX and our integrated sample preparation present a highly promising chemical approach for advancing in vivo interactomics and structural biology.

## Supporting information

As a service to our authors and readers, this journal provides supporting information supplied by the authors. Such materials are peer reviewed and may be re‐organized for online delivery, but are not copy‐edited or typeset. Technical support issues arising from supporting information (other than missing files) should be addressed to the authors.

Supporting Information

## Data Availability

Data are available at the PRIDE database with identifier PXD029034.
