# Supplementary material for: A Membrane‐Permeable and Immobilized Metal Affinity Chromatography (IMAC) Enrichable Cross‐Linking Reagent to Advance In Vivo Cross‐Linking Mass Spectrometry
Source: Angew Chem Int Ed Engl. 2022 Jan 27;61(12):e202113937. doi: 10.1002/anie.202113937 (PMC9303249; doi:10.1002/anie.202113937)
Supplement: Supplementary file 1 — Supporting Information [file ANIE-61-0-s001.pdf]

Angewandte Chemie
Eine Zeitschrift der Gesellschaft Deutscher Chemiker

Supporting Information

A Membrane-Permeable and Immobilized Metal Affinity Chromatography (IMAC) Enrichable Cross-Linking Reagent to Advance In Vivo Cross-Linking Mass Spectrometry

P.-L. Jiang, C. Wang, A. Diehl, R. Viner, C. Etienne, P. Nandhikonda, L. Foster, R. D. Bomgarden, F. Liu**

Author Contributions

P.J. and C.W. performed the experiments and data analysis. C.E., P.N., L.F., R.D.B. synthesized the cross-linker. A.D. provided *E. coli* and *B. subtilis*. P.J. and F.L. wrote the manuscript. R.D.B. and R.V. coordinated the project. F.L. developed the concept and supervised the research. All authors reviewed and edited the manuscript.

Table of Contents

Experimental Procedures 3
Materials for synthesis 3
Synthesis of dimethyl 5-(di-tert-butoxyphosphoryl) isophthalate 3
Synthesis of 5-(di-tert-butoxyphosphoryl) isophthalic acid 4
Synthesis of tBu-PhoX (tert-Butyl Disuccinimidyl Phenyl Phosphonate) 4
Materials for biochemical experiments 5
Cell culture 5
Isolation of mouse heart mitochondria 5
Cell viability assay 5
Cross-linking 5
Digestion 6
Cross-link enrichment 6
Size Exclusion Chromatography (SEC) fractionation 7
LC-MS analysis 7
Data analysis 7
Supplementary Figures 8
Figure S1 8
Figure S2 8
Figure S3 10
Figure S4 11
Figure S5 12
Figure S6 13
Figure S7 14
Figure S8 15
Figure S9 16
Table S1 17
Reference 18

Experimental Procedures

Materials for synthesis

All chemicals were commercially available and used without further purification. Solvents were anhydrous and reactions were performed under positive pressure of nitrogen. Dimethyl 5-bromoisophthalate was purchased from TCI America. Cesium carbonate, Di-tert-butyl phosphite, Lithium hydroxide and Tetrakis (triphenylphosphine)palladium (0) were purchased from Sigma-Aldrich. Flash column chromatography was performed using a Biotage Selekt. NMR spectra were recorded on a Bruker Avance AV4NEO (400 MHz) spectrometer and chemical shifts were quoted in ppm and referenced to the residual solvent signals: ^{1}H δ = 7.26 ($CDCl_3$), ^{13}C δ = 77.0 ($CDCl_3$), ^{1}H δ = 2.50 (DMSO-D_6), ^{13}C δ = 39.51 (DMSO-D_6) and 31P (162 MHz) NMR. Signal splitting patterns were described as singlet (s), doublet (d), triplet (t), quartet (q), multiplet (m), broad (br) or a combination thereof. Coupling constants (J) were measured in Hz.

Synthesis of dimethyl 5-(di-tert-butoxyphosphoryl) isophthalate

Commercially available dimethyl 5-bromoisophthalate **(1)** (10.2 g, 37.2 mmol) was dissolved in toluene and the solution was degassed with nitrogen. Di-tert-butyl phosphite **(2)** (8.4 g, 43.2 mmol, 1.16 equiv.), Cs_2CO_3 (16 g, 49.1 mmol, 1.3 equiv.) and $Pd(PPh_3)_4$ (5 mol %) was added and the reaction mixture was refluxed at 110°C for 14 h under nitrogen atmosphere. The reaction mixture was then filtered through a glass fiber filter paper to remove precipitated salts. The supernatant was evaporated in vacuo and the crude product was purified by column chromatography (Heptane/EtOAc 0 to 3:1) to obtain compound 2 in a solid state (6 g, 15.5 mmol) which gave a 42.8 % yield.

^{1}H NMR (400 MHz, $CDCl_3$) δ 1.50 (s, 18H), 3.99 (s, 6H), 8.78 (d, 2H), 8.88 (s, 1H).

^{13}C NMR (400 MHz, $CDCl_3$) δ 165.27 (C=O), 136.52, 136.41, 133.21, 130, 80, 130.64, 128.06, 83.42, 52.53, 30.51.

^{31}P NMR (162 MHz, $CDCl_3$) δ 6.7 (t)

Synthesis of 5-(di-tert-butoxyphosphoryl) isophthalic acid

Dimethyl 5-(di-tert-butoxyphosphoryl) isophthalate (4.0 g, 10.13 mmol, 1 equiv.) was dissolved in tetrahydrofuran in a round bottom flask. Next LiOH was added (0.74 g, 41.6 mmol, 3 equiv.) to a round bottom flask in a single portion. Then water was added to dissolve lithium hydroxide in the reaction mixture and stirred for 4-5 h. The reaction mixture pH was adjusted to 2-3 with acetic acid and the aqueous phase was extracted with EtOAc (2x), the combined organic phases were dried over Na_2SO_4, filtered and concentrated in vacuo to give the pure product dimethyl 5-(di-tert-butoxyphosphoryl) isophthalic acid (2.5 g, 6.98 mmol) which equals a yield of 67.3 %.

^{1}H-NMR (400 MHz, DMSO-D6*)* δ 1.50 (s, 18H), 8.78 (d, 2H), 8.88 (s, 1H).

^{13}C NMR (400 MHz, DMSO-D6) δ 166.37 (C=O), 136.44, 135.70 (C-P), 133.04, 132.15, 132.01, 83.34, 30.52. ^{31}P NMR (162 MHz, DMSO-D6*)* δ 6.94 (t)

Synthesis of tBu-PhoX (tert-Butyl Disuccinimidyl Phenyl Phosphonate)

5-(di-tert-butoxyphosphoryl) isophthalic acid (2.30 g, 6.41 mmol, 1 equiv.) was dissolved in methylene chloride, 1.81 g (17.97 mmol, 2.8 equiv.) of trimethylamine was added into the solution and then followed by addition of 3.52 g TFA. Afterwards, NHS-TFA (16.6 mmol, 2.6 equiv.) was added into the solution. The reaction mixture was stirred for 14 h at room temperature. The crude reaction was purified by column chromatography using a mixture of ethyl acetate and methanol to give pure product of tBu-Phox (1.8g, 2.3 mmol).

^{1}H-NMR (400 MHz, $CDCl_3$): 1.5 (S, 18H), 2.99 (s, 8H), 8.79-8.60 (d, 2H), 8.85(s, 1H).

^{13}C NMR (400 MHz, $CDCl_3$) δ 168.9 (C=O), 138.84, 136.77, 134.42, 126.54 (C-P), 84.32, 30.53, 25.67

^{31}P NMR (162 MHz, $CDCl_3$) δ 4.6 (t).

Materials for biochemical experiments

Chemicals for the following method sections were acquired from Sigma-Aldrich, Carl Roth or Merck unless otherwise stated.

Cell culture

HEK293T cells were cultured in Eagle's minimal essential medium (DMEM) from Gibco (DMEM, high glucose, GlutaMAX™) supplemented with 10 % (v/v) fetal bovine serum (Gibco) at 37 ºC with 5% CO2. *E. coli* were cultured in modified M9 minimal medium[1] and *B. subtilis* were cultured in C minimal medium[2] to the middle of the log growth phase at approximately OD 600 nm 0.5. Cells were harvested, washed with PBS and concentrated to OD 600 nm 100.

Isolation of mouse heart mitochondria

Hearts of adult mice were isolated and washed twice with PBS, pH 7.4 (Gibco). TH buffer (300 mM Trehalose, 10 mM KCl, 10 mM HEPES, pH 7.4) supplemented with 0.1 mg/ml BSA was used for homogenization. Homogenization was performed 25 times at 1,000 rpm on ice. Cell debris was removed by centrifugation at 400 g for 10 min. The supernatant was collected and subjected to a second centrifugation step at 800 g for 10 min to further remove cell debris. The supernatant was collected and mitochondria in the supernatant were pelleted at 11,000 g for 10 min. Mitochondria were washed three times with TH buffer without BSA. All centrifugation steps were performed at 4°C.

Cell viability assay

HEK293T cells were washed twice with PBS on culture dish. Freshly harvested cells were resuspended in PBS buffer and cell viability was measured by mixing the cells with trypan blue (Invitrogen) with 1:1 ratio (v/v). An automatic cell counter (Countess II, Invitrogen) was used to quantify the viability of cells.

Cross-linking

HEK293T cells, *E. coli* and *B. subtilis* were harvested, washed three times with PBS. HEK293T cell lysates were obtained by homogenization of freshly harvested cells in homogenization buffer (20 mM Hepes, 140 mM NaCl, 1 mM EDTA, 0.5 mM EGTA and

protease inhibitor cocktail, pH 7.4) 90 times at 1400 rpm on ice. Lysates were prepared by centrifugation at 8000 g for 10 min to remove cell debris. Freshly harvested intact cells or prepared lysates were immediately used for cross-linking. For all samples, protein concentration was adjusted to 8-10 mg/ml for cross-linking. For condition testing experiments, Crosslinking was performed using 1 mM tBu-PhoX two times, each time for 30 min at room temperature. For *in vivo* and cell lysate cross-linking using tBu-PhoX or PhoX, cross-linking was performed using 2 mM cross-linker once for 30 min at room temperature. Cross-linking reaction was quenched with 30 mM Tris-HCl pH 8.0 for 15 min at room temperature. After cross-linking, 2 mg proteins were taken as the starting material for the following procedure.

Digestion

Cross-linked proteins were digested in-solution. Proteins were denatured with 6 M urea in 50 mM TEAB, pH 8.0, reduced with 5 mM DTT at 37°C for 60 min and alkylated with 40 mM chloroacetamide at room temperature for 30 min in the dark. Protein digestion was carried out using Lysyl endopeptidase C (Wako) at an enzyme-to-protein ratio of 1:75 (w/w) at 37°C for 4 h. After diluting to 2 M urea with 50 mM TEAB, pH 8.0, the digestion was continued with trypsin (Serva) at an enzyme-to-protein ratio of 1:100 (w/w) at 37°C overnight. Samples were desalted with C8 Sep-Pak (Waters), dried under SpeedVac and stored at -20 °C until further use.

Cross-link enrichment

Digested peptide mixture was treated with 0.5% TFA for 2 h at room temperature to remove the t-butyl groups. Sample was then diluted to 80% ACN/0.1% TFA by adding 100% ACN to prepare for enrichment. Agarose Ni-NTA beads were first de-chelated by incubation in 100 mM EDTA for 30 min, and then chelated by incubation in 10 mM $FeCl_3$ for 30 min. Next, peptides were added to conditioned agarose Fe-NTA beads using a sample-to-bead ratio of 10:1 (v/v), and incubated on a shaker for 30 min. Beads were collected, loaded onto C8 StageTip (packed with YMC*GEL C8-HG Packing Material (YMC)), and washed with 80% ACN/0.1% TFA 3 times. Crosslinker-modified peptides were eluded from Fe-NTA beads to C8 StageTip with 500 mM potassium phosphate buffer (192 mM monobasic potassium phosphate and 308 mM dibasic potassium phosphate). Peptides bound to the C8 material were then washed with 0.1% FA, followed by elution with 50% ACN/0.1% FA. Eluted peptides were dried in SpeedVac for subsequent analysis.

Size Exclusion Chromatography (SEC) fractionation

Enriched cross-linker-modified peptides were fractionated by SEC using a SuperdexTM 30 Increase 3.2/300 column (GE Healthcare) on an Agilent 1260 Infinity II system. SEC was performed with a 90 min run using an isocratic flow of 0.05 ml/min. Mobile phase was 30% ACN/0.1% TFA. Five early SEC fractions were collected, dried under SpeedVac and subjected to LC-MS analysis.

LC-MS analysis

LC-MS analysis was performed using an UltiMate 3000 RSLC nano LC system coupled on-line to an Orbitrap Fusion mass spectrometer or an Orbitrap Fusion Lumos mass spectrometer (Thermo Fisher Scientific). High field asymmetric waveform ion mobility spectrometry (FAIMS) (Thermo Fisher Scientific) was enabled. Reversed-phase separation was performed using a 50 cm analytical column (in-house packed with Poroshell 120 EC-C18, 2.7 µm, Agilent Technologies) with 120 min or 180 min gradients for single-shot experiments or 180 min gradients for SEC fractions. Cross-link acquisition was performed using a LC-MS2 method. The following parameters were applied: MS resolution 120,000; MS2 resolution 30,000; charge state 3-8 enable for MS2 (unless otherwise stated); HCD energy 30, FAIMS internal stepping -50/-60/-70 V (unless otherwise stated).

Data analysis

Data analysis was performed using pLink 2.3.9[3] with the following parameters: minimum peptide length: 6; maximal peptide length: 60; missed cleavages: 3; fix modification: Cys carbamidomethyl: 57.021 Da; variable modification: Met oxidation: 15.995 Da; PhoX/tBu-PhoX cross-link: 209.972 Da; PhoX/tBu-PhoX mono-link: 227.982 Da; precursor mass tolerance: 10 ppm; fragment mass tolerance: 20 ppm. MS2 spectra were searched against full UniProt human database (retrieved in 2020). Results were reported at 1% FDR at CSM level. FDR calculation for intra-protein and inter-protein cross-links were considered separately. Mapping of cross-links were visualized in Pymol v2.5.1 using an in-house developed Python script.

Supplementary Figures

Figure S1

Membrane permeability of tBu-PhoX in A) mitochondria, B) *B. subtilis* and C) *E. coli*.

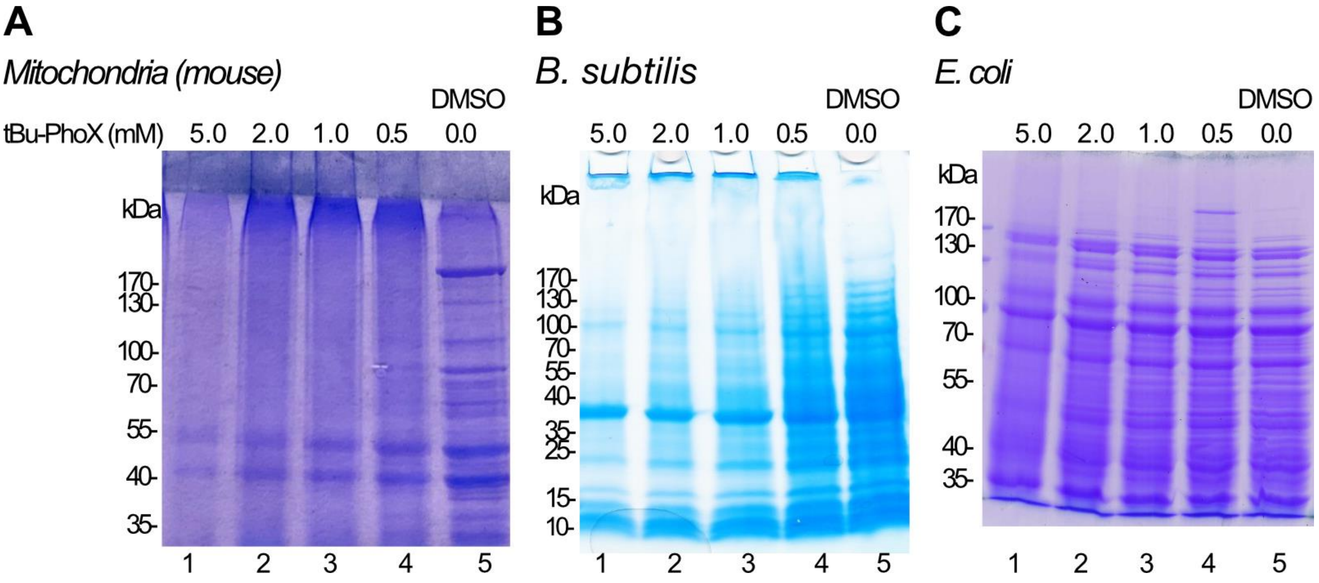

Figure S2

Optimization of sample processing and LC-MS parameters. A) Number of phosphopeptides identified from IMAC elutes. B, C) Number of cross-links and CSMs using LC-MS with different charge states enabled.

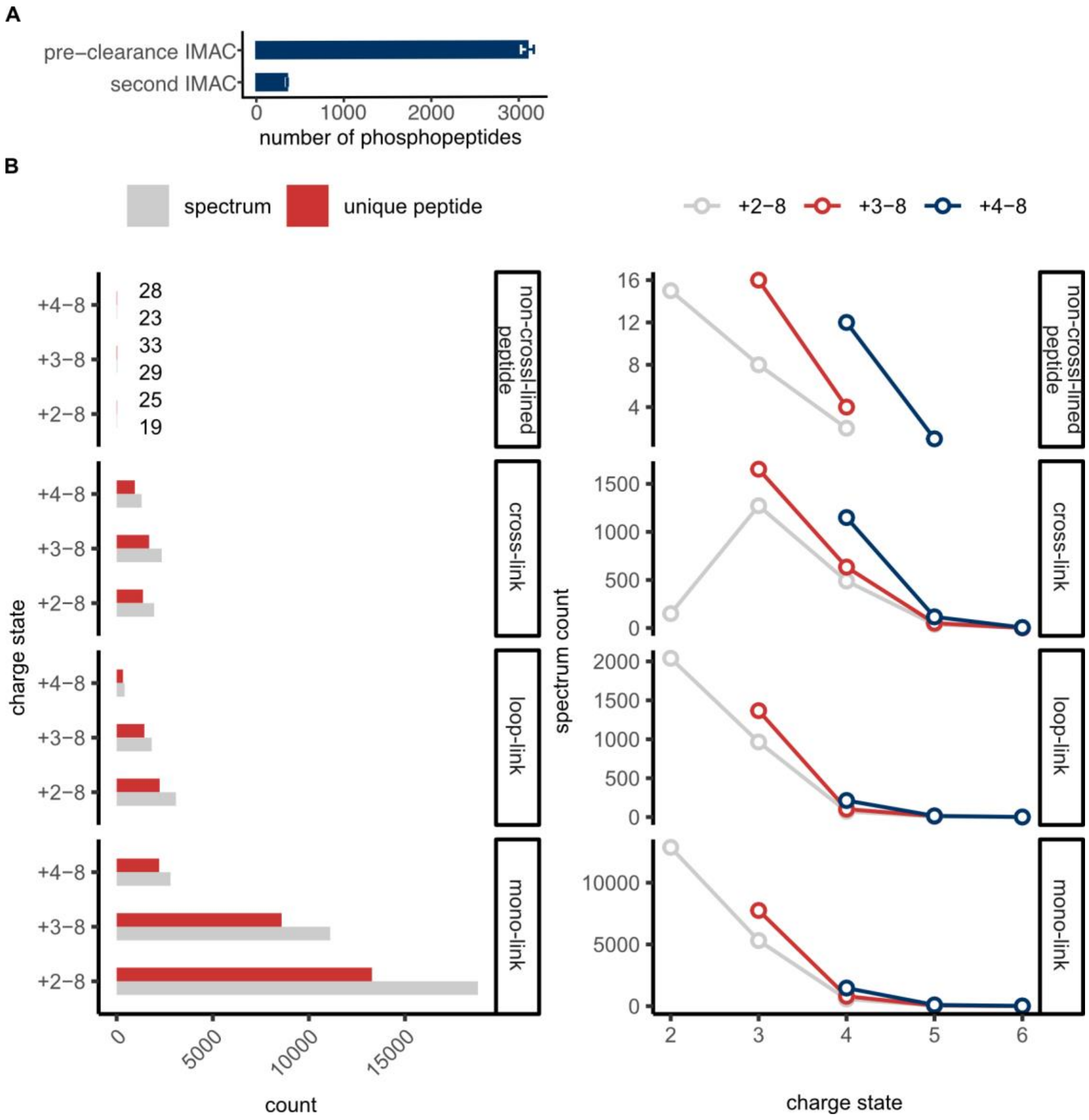

Figure S3

Optimizing FAIMS energies. A) Number of cross-links at individual CVs. B) Analysis of unique cross-links and overlap between two individual 1-CV measurements. * note the selected 2-CV combinations in Figure S3D. (C) Stacked bar plot to determine the best 3-CV combination. A first experiment (single CV measurement at -60 V) is combined with a second experiment at different CVs. Local maxima on both sides of the initial CV suggest which three CVs to combine to maximize unique cross-link identification (red) and minimize overlap between CVs (blue). Two 3-CV combinations (-50/-60/-70 V, -45/-60/-75 with * symbol), used in Figure S3D are selected based on this analysis. D) Number of cross-links and CSMs using different FAIMS CV combinations.

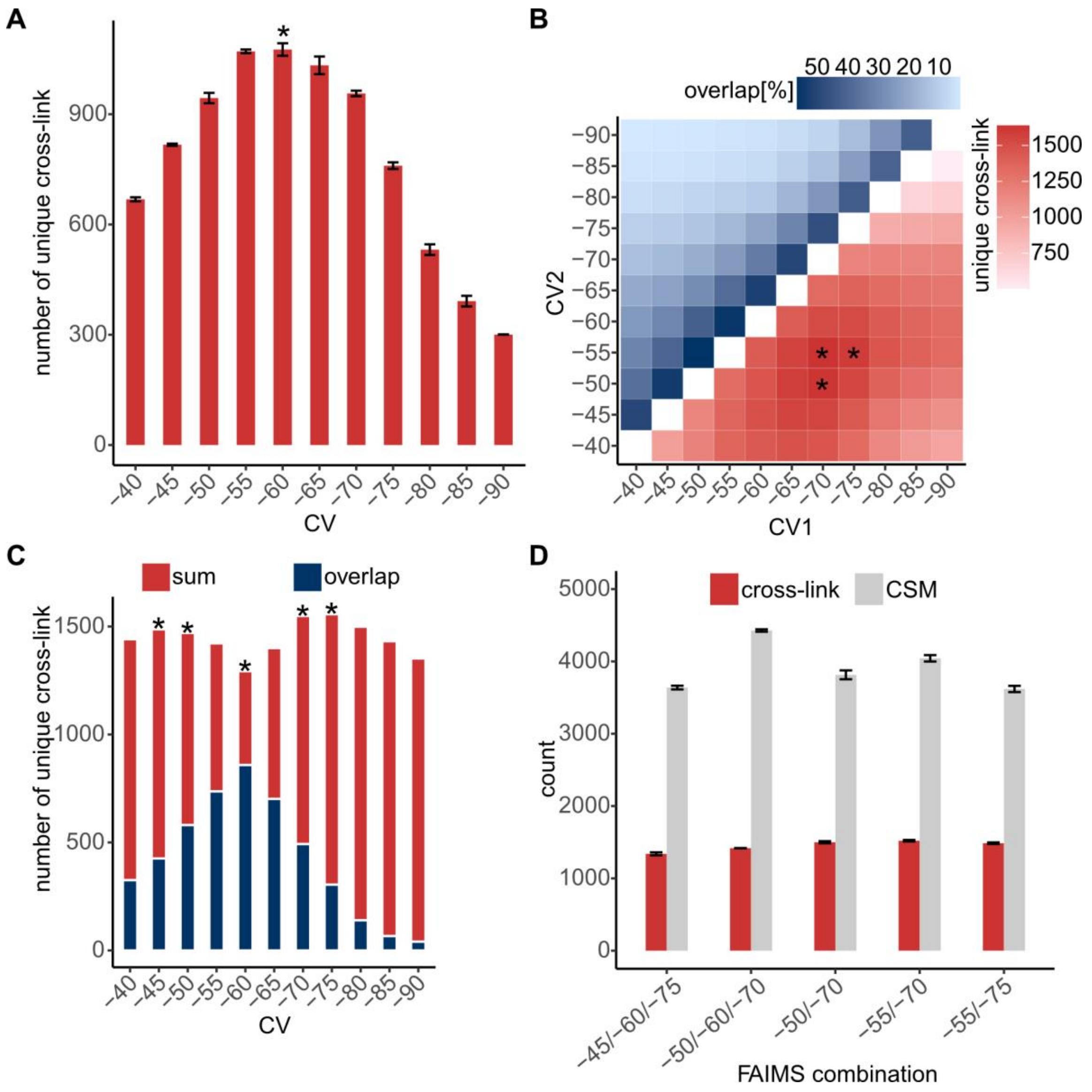

Figure S4

Cell viability assay of HEK293T cells using trypan blue staining. The viability of the cells were measured before cross-linking (0 min) and after 5, 15, 30 and 60 min cross-linking using 2 mM tBu-PhoX. The experiment was performed in triplicates.

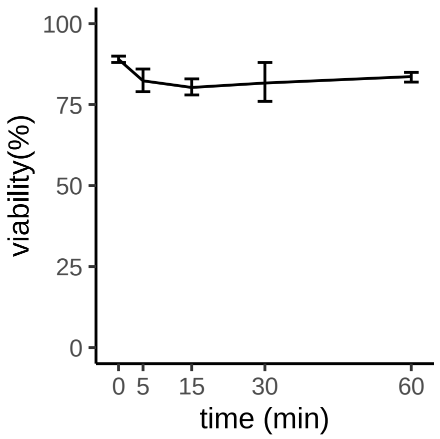

Figure S5

Number of spectra and unique peptides for each peptide type in tBu-PhoX cross-linked cells.

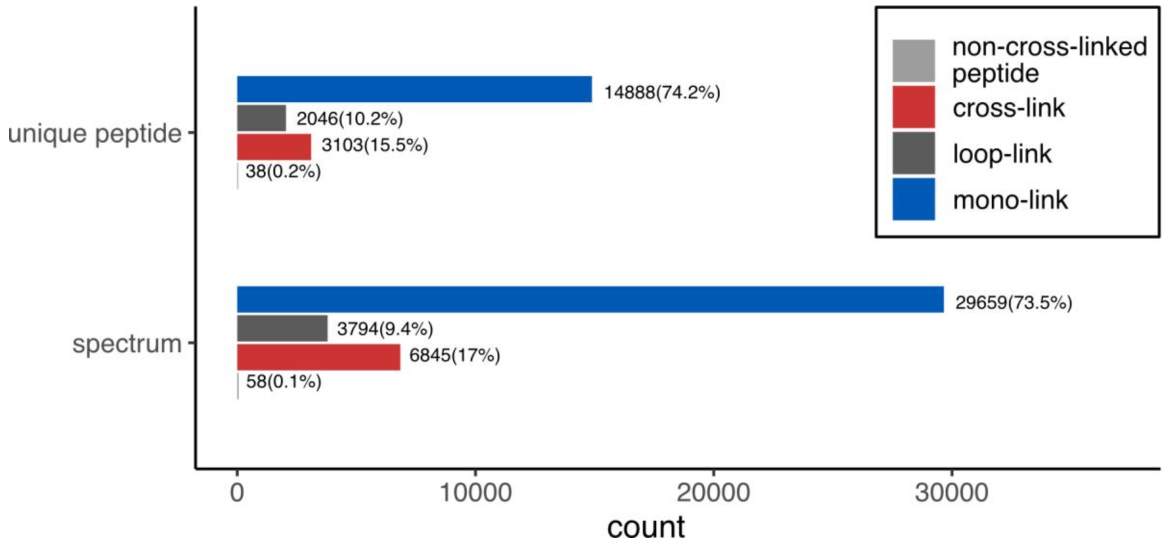

Figure S6

Distribution of cross-links *versus* mono-links in different SEC fractions. A) Percentage and number of cross-links and mono-links in different SEC fractions. B) Cosine similarity of cross-links identified in different SEC fractions.

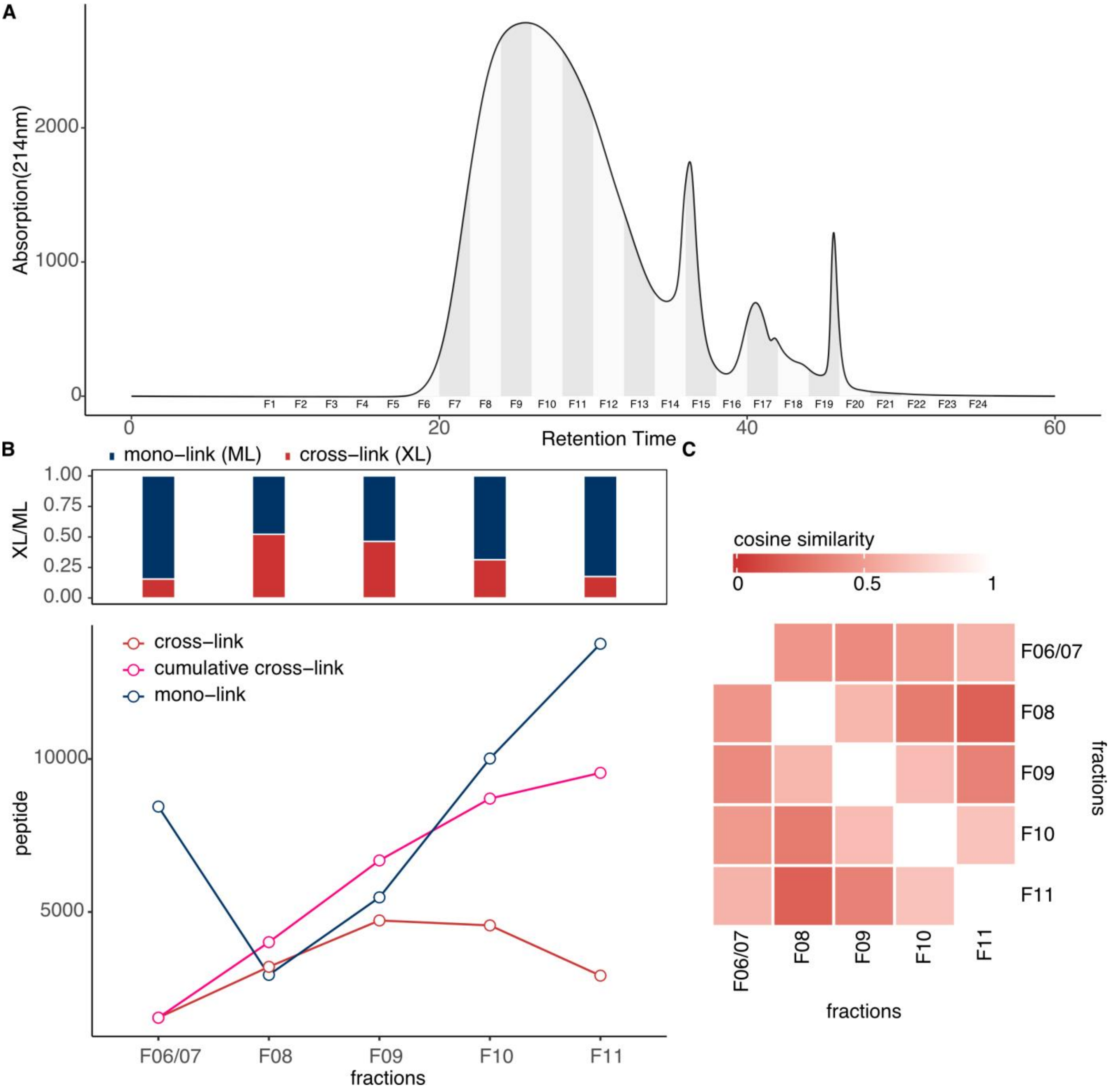

Figure S7

In vivo cross-linking of intact HEK293T cells using tBu-PhoX. A) Euler diagram shows the overlap of cross-link identifications between SEC fractions and the single-shot LC-MS measurement. B) GO terms (BP, CC, MF) from identified inter-protein cross-links using spectrum count as a quantitative measurement.

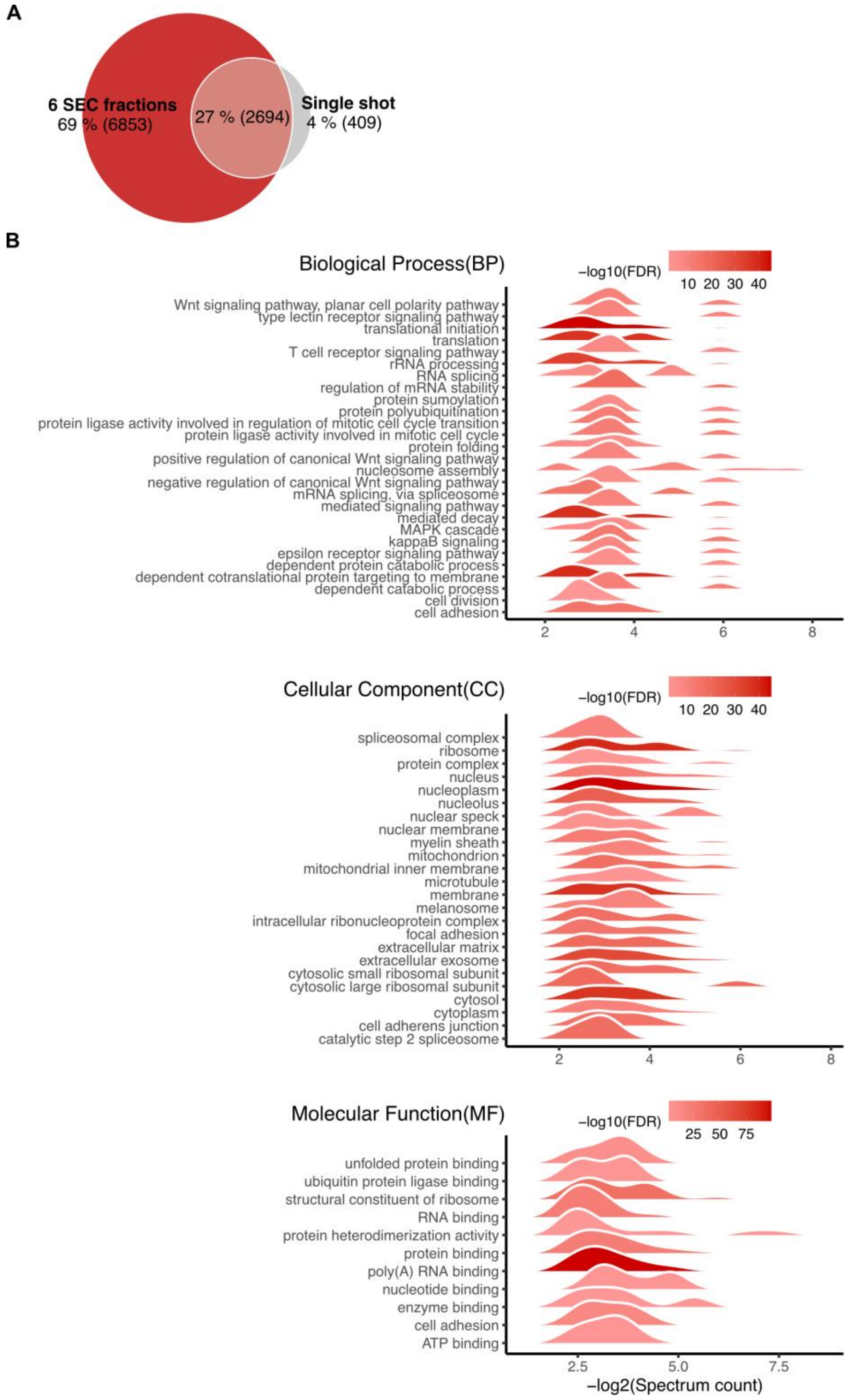

Figure S8

Visualization of cross-links on high-resolution structures of selected protein complexes.

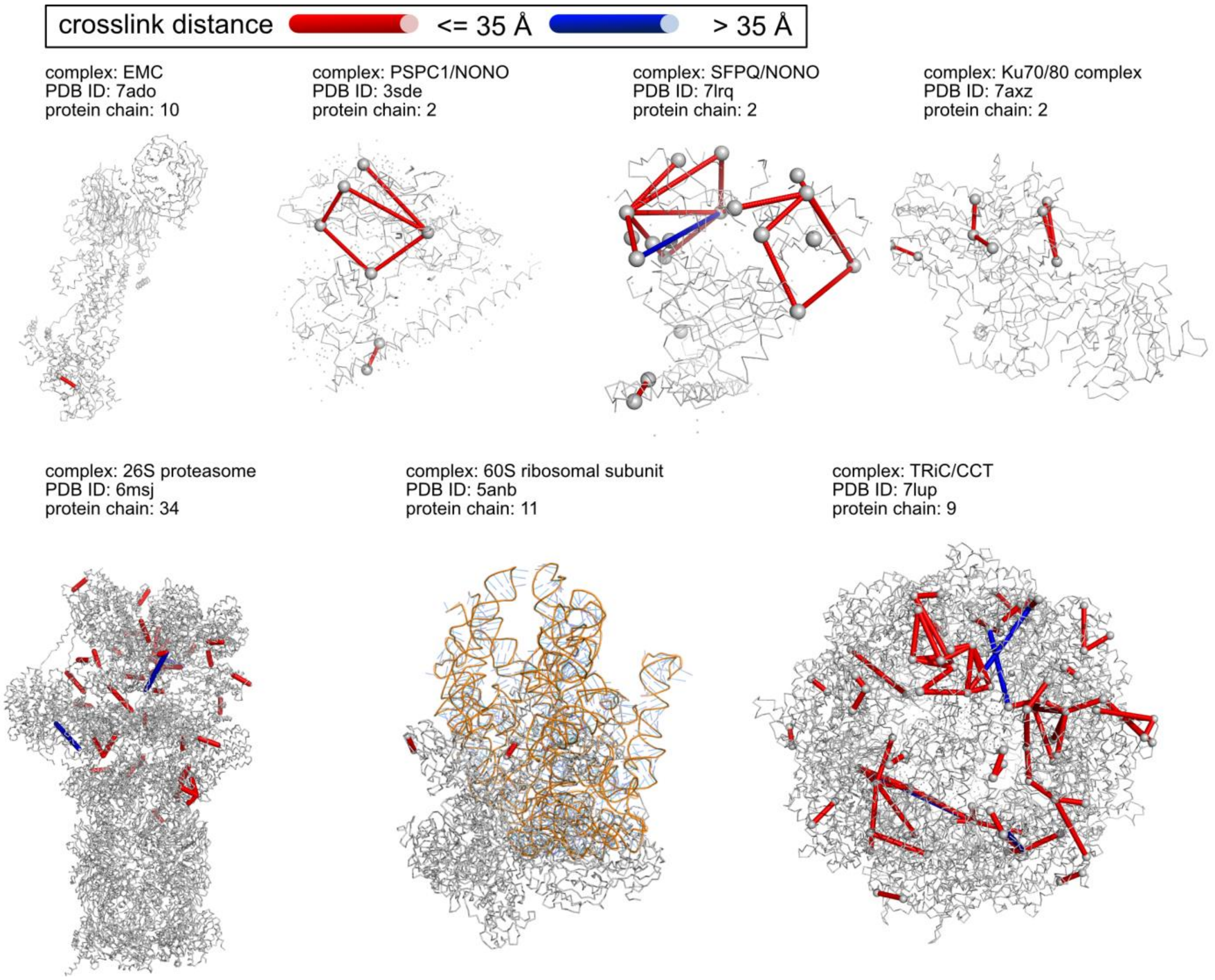

Figure S9

Comparison of PhoX and tBu-PhoX cross-linking of HEK293T cell lysates.

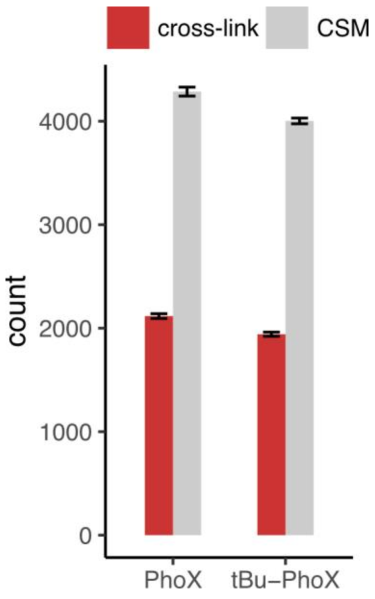

Table S1

Summary of cross-link identification and experiment information in related literatures.

Measurement time	Number of fractionations	Number of cross-links	Cross-linker	Sample	Reference
15 hours	5 (SEC)	9,547	tBu-phoX	HEK293T cell	This study
3 hours	Single shot	3,103	tBu-phoX	HEK293T cell	This study
15 hours	5 (SEC)	9,393	tBu-phoX	HEK293T cell lysate	This study
32.5 days	2 samples × 9 SCX × 10 hSAX	5,655(DSSO) 6,923(BS3)	DSSO, BS3	E. coli lysate	Rappsilber, 2021 [4]
60 hours	20 (SCX)	11,999	DSSO	Mouse synaptosome	Smit, 2020 [5]
15 days	9 (SCX)× 15 (hSAX)	10,552	DSSO	Mycoplasma	Rappsilber, 2020 [6]
5 days	18 (SEC)	7,436	DSBU	Drosophila embryo extracts	Sinz, 2019 [7]
100 hours	50 (SCX)	~8,700	DSSO	U2OS nuclei	Heck, 2018 [8]
12 hours	6 (SCX)	575	DSSO	E. coli lysate	Heck, 2017 [9]

Reference

[1] A. Diehl, Y. Roske, L. Ball, A. Chowdhury, M. Hiller, N. Moliere, R. Kramer, D. Stoppler, C. L. Worth, B. Schlegel, M. Leidert, N. Cremer, N. Erdmann, D. Lopez, H. Stephanowitz, E. Krause, B. J. van Rossum, P. Schmieder, U. Heinemann, K. Turgay, U. Akbey, H. Oschkinat, *Proc Natl Acad Sci U S A* **2018**, *115*, 3237-3242.

[2] F. M. Commichau, K. Gunka, J. J. Landmann, J. Stulke, *J Bacteriol* **2008**, *190*, 3557-3564.

[3] Z. L. Chen, J. M. Meng, Y. Cao, J. L. Yin, R. Q. Fang, S. B. Fan, C. Liu, W. F. Zeng, Y. H. Ding, D. Tan, L. Wu, W. J. Zhou, H. Chi, R. X. Sun, M. Q. Dong, S. M. He, *Nat Commun* **2019**, *10*, 3404.

[4] S. Lenz, L. R. Sinn, F. J. O'Reilly, L. Fischer, F. Wegner, J. Rappsilber, *Nat Commun* **2021**, *12*, 3564.

[5] M. A. Gonzalez-Lozano, F. Koopmans, P. F. Sullivan, J. Protze, G. Krause, M. Verhage, K. W. Li, F. Liu, A. B. Smit, *Sci Adv* **2020**, *6*, eaax5783.

[6] F. J. O'Reilly, L. Xue, A. Graziadei, L. Sinn, S. Lenz, D. Tegunov, C. Blotz, N. Singh, W. J. H. Hagen, P. Cramer, J. Stulke, J. Mahamid, J. Rappsilber, *Science* **2020**, *369*, 554-557.

[7] M. Gotze, C. Iacobucci, C. H. Ihling, A. Sinz, *Anal Chem* **2019**, *91*, 10236-10244.

[8] D. Fasci, H. van Ingen, R. A. Scheltema, A. J. R. Heck, *Mol Cell Proteomics* **2018**, *17*, 2018-2033.

[9] F. Liu, P. Lossl, R. Scheltema, R. Viner, A. J. R. Heck, *Nat Commun* **2017**, *8*, 15473.
